# The Reform of Club Practice

**Published:** 1912-04-13

**Authors:** 


### The Hospital

The Workers' Newspaper of Administrative Medicine and Institutional Life, Administration, National Insurance and Health.

No. 1344, Vol. LII. SATURDAY, APRIL 13, 1912. PRICE ONE PENNY.

### THE REFORM OF CLUB PRACTICE.

Last week attention was invited in these columns to the lack of information concerning the intentions and deliberations of the Insurance Commissioners and to the steady approach of the fateful month of July. So far as the Commission is concerned, no more light has been shed as yet upon the situation thus created, and it is still quite uncertain what the course and the upshot of the negotiations may be that are presumably being carried on. It would be unreasonable to demand that every phase of the dealings between the Commission and the representatives of the medical profession should be immediately made public, for such a facing of the cards might embarrass the players. But this continued and absolute silence is carrying the matter to extremes, and is not only regrettable in itself but also by no means a good augury of ultimate agreement.

Meanwhile all that the profession and the hospital public can do is to note very keenly in what way the straws are blowing. Possibly this very keenness may lead observers to form an exaggerated impression of the meaning of things that are in themselves trivial. But, for ourselves, we confess that the scheme of medical defence in connection with contract practice, issued a week ago from headquarters to the divisions of the British Medical Association for consideration, is timed so aptly that we cannot regard it as devoid of significance. The documents issued by the Council of the Association have been labelled "private and confidential," but their contents have been public property for some days. Their provisions form the outline of an organisation which will come into existence if and when the negotiations with the Insurance Commissioners prove abortive. This may be, no doubt, merely a precautionary measure, but unless the Council is playing a game of bluff, which we decline to credit, it certainly seems that no favourable outcome of the negotiations is believed to be at all probable.

So much for what will be generally read into the issue of this scheme; let us turn for a moment to the firmer ground of the provisions themselves. The object in view is the establishment of a contract medical service by a united medical profession outside the jurisdiction of the Insurance Commissioners. In other words, it is the counterblast of the British Medical Association to the Chancellor's threats of a suspension of medical benefits under the Act. The latter has boasted that a revolt of the profession against the terms offered in the Act was foreseen and provided for. He and his colleague, Mr. Masterman, have openly rated the doctors for refusing to accept those terms, and have explained that such a refusal will merely place the profession in much larger numbers and to a much greater degree under the thumb of the greedy Friendly Societies. For the "suspension of medical benefit" simply means that the Insurance Commission will pay over to the Societies a sum—probably six shillings per insured person per annum—which would have been available for the remuneration of medical services under the Act. The upshot is imagined to be that the Societies will continue to exploit the jealousies and disunion of the profession by paying the lowest sum (say, four shillings or four-and-six) for which the more indigent of the doctors can be induced to accept the work, and will pocket the difference for their own funds.

This pretty scheme is very ingenious, and at first sight it might be thought better to accept the six shillings *faute de mieux*. But is it really certain that the Friendly Societies could continue their old sweating rates in the changed circumstances? Here and there they might succeed; but the Act itself and all the discussion it has given rise to have so consolidated the profession that in the main the attempt would probably fail.

Here the scheme of the British Medical Association finds its scope. On terms which will be satisfactory to itself the profession offers to undertake contract practice on a national scale. If the Societies or the Commissioners choose to accept those terms, well and good; if they do not, the Insurance Act becomes waste paper provided the profession sticks firmly to the British Medical Association's defence scheme and refuses to be cajoled by pettifogging politicians. The profession secures those important points—a wage limit is one—over which Mr. Lloyd George tricked them in the Lobby; it secures a living wage, and one upon which it can both maintain its own self-respect and obtain that of its patients. The latter, in their turn, secure medical attendance of a more efficient kind than they have ever hitherto had, and in securing this they secure a very great deal more than ninepence for a good deal less than fourpence.

